# Effect of Prolonged Moderate Exercise on the Changes of Nonneuronal Cells in Early Myocardial Infarction

**DOI:** 10.1155/2015/265967

**Published:** 2015-07-22

**Authors:** Barbara Rinaldi, Francesca Guida, Anna Furiano, Maria Donniacuo, Livio Luongo, Giulia Gritti, Konrad Urbanek, Giovanni Messina, Sabatino Maione, Francesco Rossi, Vito de Novellis

**Affiliations:** Department of Experimental Medicine, Division of Pharmacology, The Second University of Naples, Via Costantinopoli 16, 80138 Naples, Italy

## Abstract

Myocardial infarction (MI) is one of the leading causes of death in developed countries and it is characterized by several associated symptomatologies and poor quality of life. Recent data showed a possible interaction between infarction and brain inflammation and activity. Previous studies have demonstrated the beneficial effect of exercise training on deterioration in cardiac function after MI. In this study we analyzed in sedentary and trained rats the microglia and astrocytes 48 hours after MI in PVN, thalamus, prefrontal cortex, and hippocampus through immunofluorescence approach. We found significant changes in specific microglia phenotypes in the brain areas analyzed together with astrocytes activation. Prolonged exercise normalized these morphological changes of microglia and astrocytes in the prefrontal cortex, hippocampus, and thalamus but not in the PVN. Our data suggest that there is an early brain reaction to myocardial infarction induction, involving nonneuronal cells, that is attenuated by the prolonged exercise.

## 1. Introduction

Myocardial infarction (MI) is one of the leading causes of death in developed countries and it is characterized by several associated symptomatologies and a poor quality of life [1].

Features of the MI include an increase of cardiac sympathetic nerve activity to elicit inotropic positive response for the maintenance of cardiac output; this compensatory mechanism plays a major role in the progression of heart failure [2].

Recent data showed the role of the hypothalamic paraventricular nucleus (PVN), a key neurohumoral integrative nucleus in the brain, responsible for the connections to sympathetic preganglionic motor neurons [3, 4]. Moreover, high percentage of MI patients undergo late depressive state. Brain inflammation, driven by nonneuronal cells such as astrocytes and microglia, has been demonstrated to be associated with several neurological diseases including depression and anxiety [5], with neuropsychiatric pathologies such as mood disorders and psychosis [6], and with neurodegenerative diseases such as Parkinson, Alzheimer, and Huntington diseases [7–9]. Indeed, all these pathologies are also associated with plastic changes which involve the activation of glia and microglia in the early sensitization and the subsequent neuronal suffering. Moreover, glial and microglial cells have been shown to be associated also with the chronicity of pain that could be consequence of heart failures such as myocardial infarction [10–12]. Recent reports have analyzed the possible changes in microglial phenotypes after myocardial infarction at different time stages, demonstrating a microglial cytoskeletal rearrangement in a late phase after MI induction in the PVN whereas no significant differences were observed in the cortex [13, 14]. This microglia activation seems to be mediated by the P2X7 receptor [15] and also an attenuation of microglial and neuronal activation in the brain by ICV minocycline following myocardial infarction has been reported [16]. Previous studies have demonstrated the beneficial effect of exercise training on deterioration in cardiac function after MI [17, 18]. In this study we analyzed in sedentary and trained rats the microglia and astrocytes 48 hours after MI in PVN, thalamus, prefrontal cortex, and hippocampus through immunofluorescence approach.

## 2. Materials and Methods

### 2.1. Animals and Experimental Design

All the experimental procedures were approved by the Animal Ethics Committee of the Second University of Naples. Animal care was in compliance with Italian (D.L. 116/92) and European Community (E.C. L358/1 18/12/86) guidelines on the use and protection of laboratory animals. All efforts were made to minimize animal suffering and to reduce the numbers of animals used.

### 2.2. Training Protocol

Sixty rats (225–250 g; *n* = 60; Harlan Italy) were randomly assigned to two main groups: sedentary (SED; *n* = 30) and exercise trained (TR; *n* = 30). The training protocol was performed as previously described [19]. Briefly, trained rats were acclimated to training by walking at a speed of 10 min/day on a treadmill for 2 weeks (Panlab/Harvard Apparatus Treadmills, Holliston, MA, USA). From week 3, speed and time of running were gradually increased (30 m/min, 45 min/day, 5 days/week, for 6 weeks). The sedentary rats remained in the cages for all the duration of the training protocol.

### 2.3. Surgical Procedure

Twenty-four hours from the last session of exercise training 20 rats of both experimental groups were undergoing myocardial infarction by surgical occlusion of the left anterior descended (LAD) coronary artery, according to previous described procedures [20]. Briefly, after induction with intraperitoneal injection of ketamine hydrochloride (100 mg/kg) and xylazine (2.5 mg/kg) supplemented as needed, the rats were intubated and ventilated with room air by a small animal ventilator (Harvard Apparatus, Model 623). After performing the thoracotomy in the third and fourth intercostals spaces, the pericardium was incised and a 6-0 silk suture (Johnson & Johnson) was placed around the proximal portion of the left coronary artery. The chest was closed with a 4-0 silk purse-string suture. The rats were awoken within 5–30 minutes postoperatively. Following MI, the animals remained supervised until fully conscious. Two days after MI all the animals were sacrificed.

### 2.4. Immunohistochemistry and Immunofluorescence

#### 2.4.1. For Cardiac Staining

Formalin-fixed, paraffin-embedded myocardial samples were cut in 5 *μ*m thick sections and stained with hematoxylin and eosin. For fluorescence imaging, tissue sections were deparaffinized and labelled with ApoAlert DNA Fragmentation Assay Kit (Clontech). Subsequently, samples were incubated with an anti-*α*-sarcomeric actin (Sigma) antibody followed by TRITC-conjugated secondary antibody (*Jackson Immuno, West Baltimore Pike, West Grove, PA, USA*). Samples were analyzed with a Leica DM 5000B microscope and a Zeiss LSM 700 confocal microscope.

#### 2.4.2. For Brain Staining

Under pentobarbital anesthesia (50 mg/kg, i.p.), animals were transcardially perfused with saline solution followed by 4% paraformaldehyde in 0.1 M phosphate buffer. The brains were excised, postfixed for 3 h in the perfusion fixative, cryoprotected for 72 h in 30% sucrose in 0.1 M phosphate buffer, and frozen in* optimal cutting temperature* (O.C.T.) embedding compound. Transverse sections (20 *μ*m) were cut using a cryostat and thaw-mounted onto glass slides. Slides were incubated overnight with primary antibody solutions for the microglial cell marker Iba-1 (rabbit anti-ionized calcium binding adapter molecule-1; 1 : 1000;* Wako Chemicals, Germany*) and GFAP (rabbit polyclonal antiglial fibrillary acidic protein; 1 : 1000;* Dako Cytomation, Denmark*), according to previously reported protocols [21, 22]. Possible nonspecific labeling of mouse secondary antibody was detected by using secondary antibody alone. Following incubation, sections were washed and incubated for 2 h with secondary antibody solution (donkey anti-rabbit or IgG-conjugated Alexa Fluor 488; 1 : 1000;* Molecular Probes, USA*). Slides were washed, coverslipped with VECTASHIELD mounting medium (*Vector Laboratories, USA*), and visualized under a* Leica fluorescence microscope*.

### 2.5. Quantitative Image Analysis

The number of cells positive for Iba-1 or GFAP was determined within a box measuring 2 × 10^4^ *μ*m^2^ that was placed in the lateral, central, and medial areas of cortex, hippocampus, thalamus, and hypothalamic paraventricular nucleus. Eight sections were assessed from one animal and three animals were used for each group. To avoid cell overcounting, only DAPI-counterstained cells were considered as positive profiles. Iba-1 and GFAP-positive cells were identified as resting (with small somata bearing long, thin, and ramified processes), activated microglia and astrocytes (with hypertrophy together with retraction of processes to a length shorter than the diameter of the somata), or dystrophic microglia (no dystrophic astrocytes were detected). Dystrophic microglia was recognized by debris consisting of several cells displaying fragmented processes and an irregularly shaped cell body as previously demonstrated in humans [23].

## 3. Results

### 3.1. Infarct Evaluation

**Figure 1:**
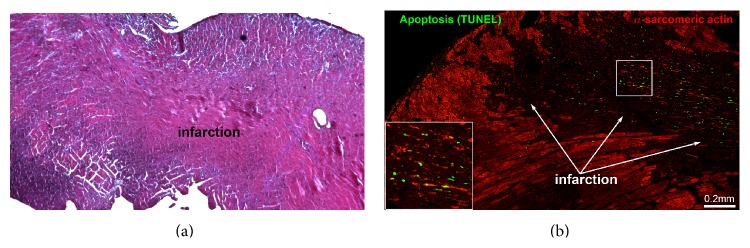
Infarct evaluation. Myocardial infarction 48 hours after coronary artery ligation. Hematoxylin and eosin staining with hypereosinophilic cardiomyocytes and early inflammatory infiltrates (upper panel). Confocal image of the infarcted heart (lower panel) showing apoptosis (TUNEL, green) and the disintegration of structural protein *α*-sarcomeric actin (red) in cardiomyocytes present in the infarcted area. The square represents a high magnification of the TUNEL positive profiles.

Forty-eight hours after coronary artery ligation, the presence of myocardial infarction was confirmed by histological analysis. Typical features of early myocardial infarction were present in H&E-stained sections. This was confirmed by the detection of apoptotic cardiomyocytes with a terminal deoxynucleotide transferase- (TdT-) mediated dUTP nick-end labeling (TUNEL) assay and confocal microscopy (Figure 1).

### 3.2. Activation of Microglia and Astrocytes

#### 3.2.1. Cortex

**Figure 2:**
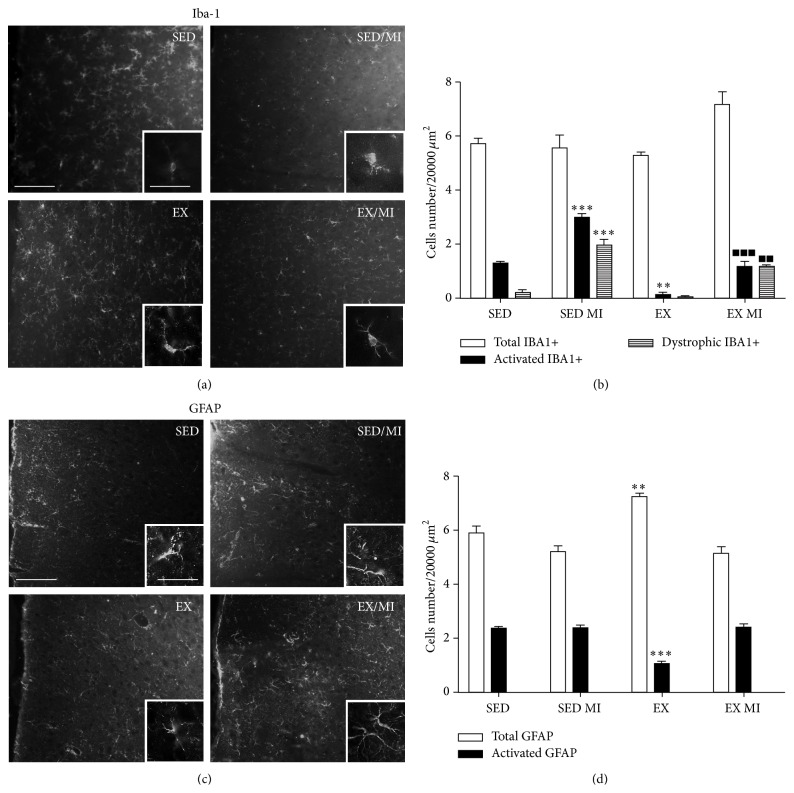
Effect of prolonged exercise on MI induced microglia and astrocyte morphological changes in the prefrontal cortex. 48 h MI induced significant changes in hypertrophic (2.20 ± 0.32) and senescent (1.97 ± 0.2) (dystrophic-like) microglia in the prefrontal cortex as compared to the sedentary group without MI (1.29 ± 0.02 and 0.19 ± 0.12). Both phenotypes were significantly reduced by the prolonged exercise (1.16 ± 0.19 and 1.13 ± 0.07) (a, b). 48 h MI did not modify the total (5.88 0.27) or reactive (2.36 0.05) astrocyte number in the prefrontal cortex, whereas exercise alone enhanced the resting/hypertrophic astrocyte ratio (7.25 ± 0.09 and 1 ± 0.12) (c, d). Data are presented as mean ± SEM. ANOVA followed by Tukey post hoc test was used for statistical analysis. Three animals were used for each experimental group. ^*∗*^ *P* < 0.05, ^*∗∗*^ *P* < 0.01, and ^*∗∗∗*^ *P* < 0.001 versus sedentary group; ^▪^ *P* < 0.05, ^▪▪^ *P* < 0.01, and ^▪▪▪^ *P* < 0.001 versus sedentary group with MI. Scale bars 100 and 25 *μ*m for panoramic and inset image, respectively.

MI procedure did not change the number of both total Iba-1 positive cells in the cortex. However, MI animals showed a significant increase of the number of activated as well as dystrophic microglia cells in the same area. These effects were significantly prevented by applying the exercise training protocol (Figures 2(a) and 2(b)). No changes were observed in astrocytes following MI surgery. Interestingly, the exercise alone exhibited significantly effects on cells morphological changes, as compared with sedentary animals. In particular, it reduced the number of activated microglia cells and modulated the number of astrocytes, by increasing the total number of GFAP-positive cells, and reduced the number of hypertrophic cells (Figures 2(c) and 2(d)). 

**Figure 3:**
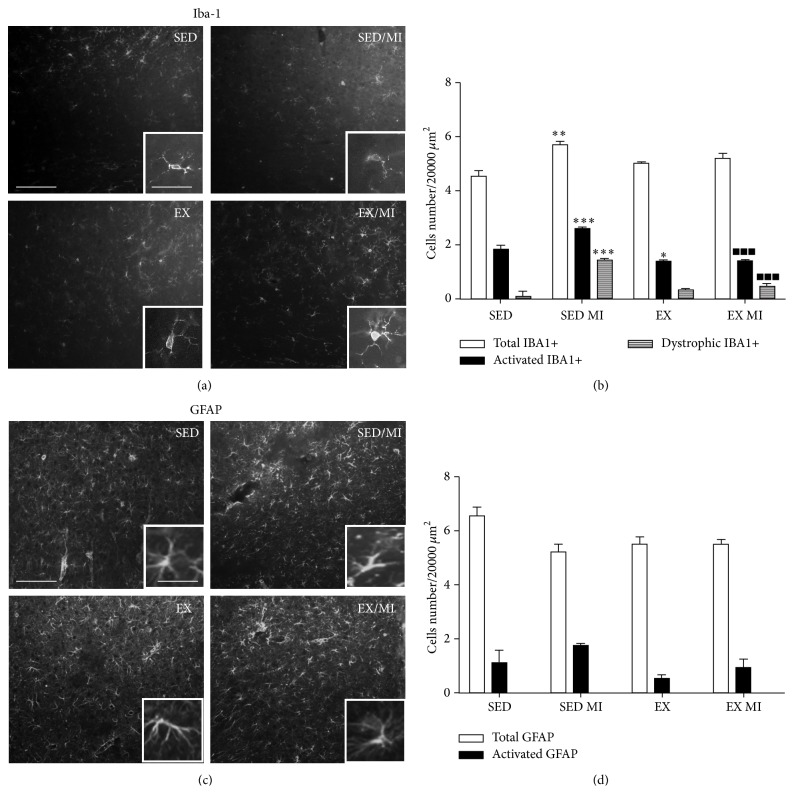
Effect of prolonged exercise on MI induced microglia and astrocyte morphological changes in the hippocampus. 48 h MI induced significant changes in hypertrophic 2.6 ± 0.05 and senescent 1.4 ± 0.03 (dystrophic-like) microglia in the hippocampus as compared to the sedentary group without MI (1.80 ± 0.19 and 0.11 ± 0.19). Both phenotypes were significantly reduced by the prolonged exercise (1.4 ± 0.04 and 0.47 ± 0.1) (a, b). 48 h MI did not modify the total or reactive astrocyte number in the hippocampus (5.2 ± 0.26 and 1.74 ± 0.07) (c, d). Data are presented as mean ± SEM. ANOVA followed by Tukey post hoc test was used for statistical analysis. Three animals were used for each experimental group. ^*∗*^ *P* < 0.05, ^*∗∗*^ *P* < 0.01, and ^*∗∗∗*^ *P* < 0.001 versus sedentary group; ^▪^ *P* < 0.05, ^▪▪^ *P* < 0.01, and ^▪▪▪^ *P* < 0.001 versus sedentary group with MI. Scale bars 100 and 25 *μ*m for panoramic and inset image, respectively. Scale bars 100 and 25 *μ*m for panoramic and inset image, respectively.

*Hippocampus*. We observed a significant increase of total, activated, and dystrophic microglia cells in the hippocampus of MI rats. The exercise counteracted the microglia activation and reduced the number of dystrophic cells, without affecting the total cells number in the same area. Moreover, the exercise reduced per se the number of activated microglia as compared with sedentary animals (Figures 3(a) and 3(b)). No changes in astrocytes morphology were observed (Figures 3(c) and 3(d)).

#### 3.2.2. Thalamus

**Figure 4:**
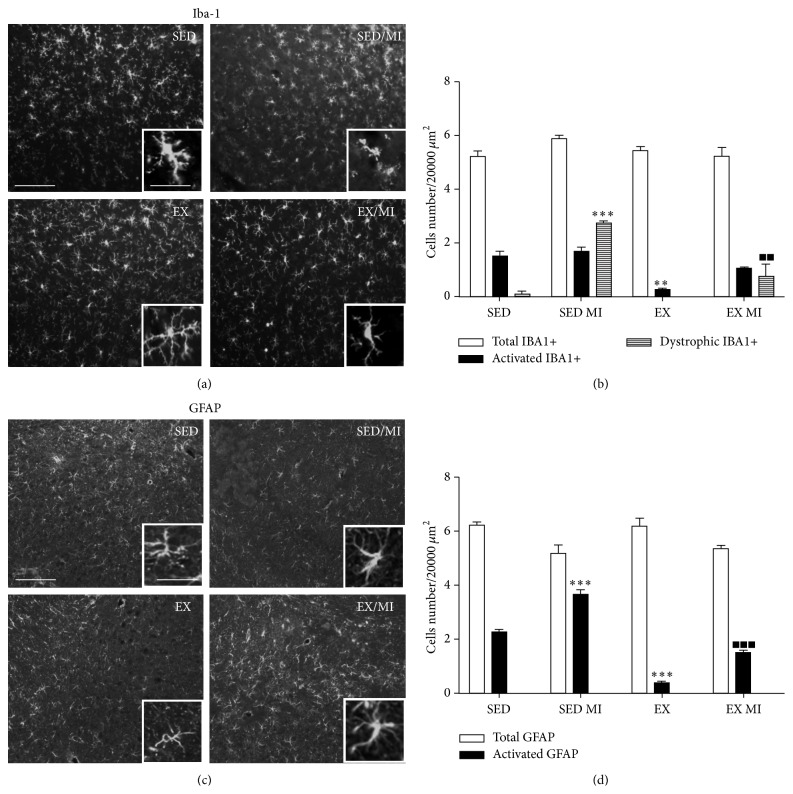
Effect of prolonged exercise on MI induced microglia and astrocyte morphological changes in the thalamus. 48 h MI induced significant changes in the senescent (2.7 ± 0.12) (dystrophic-like) microglia in the thalamus as compared to the sedentary group without MI (0.11 ± 0.11). Both phenotypes were significantly reduced by the prolonged exercise 0.7 ± 0.44 (a, b). 48 h MI induced increased number of reactive astrocytes (3.6 ± 0.19) as compared to the sedentary group without MI (2.25 ± 0.08). Exercise significantly reduced the number of reactive astrocytes in MI animals (1.5 ± 0.08) (c, d). Data are presented as mean ± SEM. ANOVA followed by Tukey post hoc test was used for statistical analysis. Four animals were used for each experimental group. ^*∗*^ *P* < 0.05, ^*∗∗*^ *P* < 0.01, and ^*∗∗∗*^ *P* < 0.001 versus sedentary group; ^▪^ *P* < 0.05, ^▪▪^ *P* < 0.01, and ^▪▪▪^ *P* < 0.001 versus sedentary group with MI. Scale bars 100 and 25 *μ*m for panoramic and inset image, respectively. Scale bars 100 and 25 *μ*m for panoramic and inset image, respectively.

The MI induction did not change the number of the total or activated microglia cells in the thalamus as compared with control. However, the number of dystrophic microglia dramatically increased in MI animals 2 days after surgery. Exercise trained animals showed a significantly attenuated number of dystrophic microglia induced by MI by more than 50%. Interestingly, the exercise erased per se the number of activated microglia in sedentary rats (Figures 4(a) and 4(b)). The total numbers of astrocytes counted in the thalamus of the different treatment groups were similar. However, MI induced an increase in the number of hypertrophic GFAP-positive cells that were significantly reduced in the trained mice. Moreover, the exercise training abolished the number of hypertrophic astrocytes in sedentary rats (Figures 4(c) and 4(d)).

#### 3.2.3. Hypothalamic Paraventricular Nucleus

**Figure 5:**
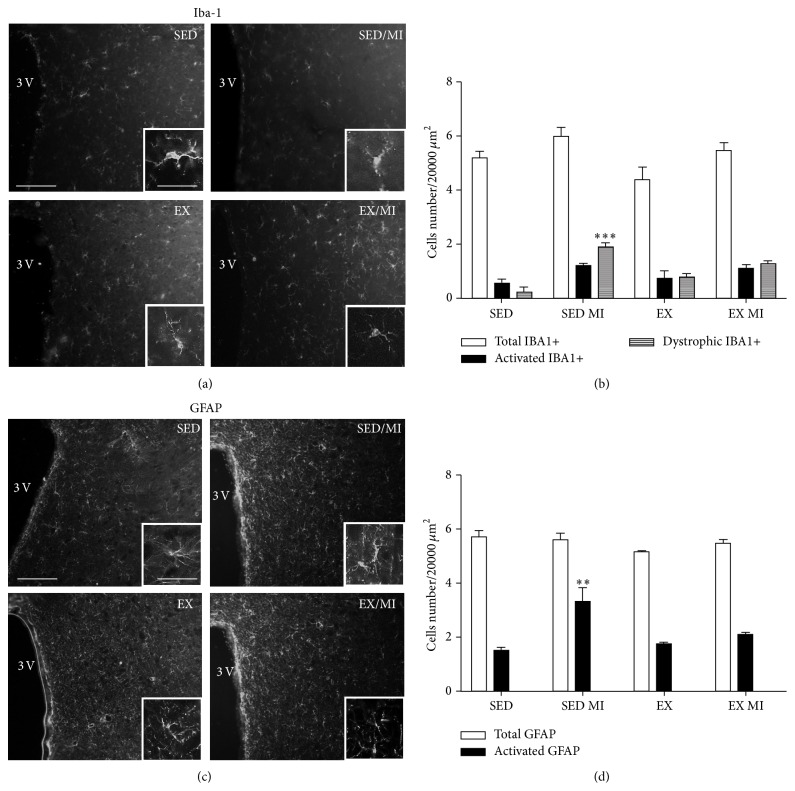
Effect of prolonged exercise on MI induced microglia and astrocyte morphological changes in the PVN. 48 h MI induced significant changes in the senescent (1.8 ± 0.1) (dystrophic-like) microglia in the PVN as compared to the sedentary group without MI (0.2 ± 0.1) (a, b). 48 h MI induced increased number of reactive astrocytes in the PVN (3.3 ± 0.51) versus control (1.5 ± 0.09). (c, d). Data are presented as mean ± SEM. ANOVA followed by Tukey post hoc test was used for statistical analysis. Four animals were used for each experimental group. ^*∗*^ *P* < 0.05, ^*∗∗*^ *P* < 0.01, and ^*∗∗∗*^ *P* < 0.001 versus sedentary group. Scale bars 100 and 25 *μ*m for panoramic and inset image, respectively.

MI and sedentary rats did not differ in the number of total microglia as well as astrocytes in the PVN. Furthermore, MI surgical procedure did not change the number of activated microglia cells found in the control animals (~10%). However, MI rats showed an increased number of dystrophic microglia cells as well as hypertrophic astrocytes which were not affected by exercise training (Figures 5(a)–5(d)).

## 4. Discussion

This study provides the first evidence that the possible changes in glia and microglia brain areas after myocardial infarction could be, at least in part, prevented by the prolonged moderate exercise. In this study we analyzed the morphological changes of the microglia and astrocytes in four brain areas involved in the integration of external emotional stimuli, learning and memory, sensorial perception, and metabolism, before and 48 hours after myocardial infarction and with or without 8-week training protocol.

In particular, we observed that in the prefrontal cortex the 48 h MI induced an increased number of hypertrophic and dystrophic-like microglia in sedentary mice. Both microglia phenotypes were significantly reduced in the prefrontal cortex in the 48 h MI trained group. No significant changes in the hypertrophic/resting astrocytes ratio were observed in the prefrontal cortex in all experimental groups except for the trained mice without MI in which we found an increase and decrease in the total and hypertrophic astrocytes, respectively. In the thalamus and hippocampus, which represent key brain areas in the sensorial and pain-associated synaptic plasticity, we observed an increased number of dystrophic microglia. Furthermore, in the thalamus, microglial changes were also accompanied by an augmented number of hypertrophic astrocytes, in the MI group as compared to the sedentary animals. The prolonged exercise significantly reduced both the dystrophic microglia and reactive astrocytes induced by MI. Finally, in the PVN we observed increased number of dystrophic microglia and hypertrophic astrocytes in the sedentary animals that were not changed by the exercise. The latter data are interesting also regarding the role of the PVN which represents an important neuroendocrine and preautonomic output nucleus and is considered as the important central site for integration of sympathetic nerve activity [3, 4]. This could be explained, at least in part, assuming that the effect of exercise is mainly acting on those brain areas involved in the sensorial and rewarding processes such as thalamus and cortical areas rather than those brain structures directly involved in the interface between CNS and periphery. Intriguingly, previous data showed no changes in activated microglia in the PVN and cortex 24 hours after myocardial infarction induction [13, 14]. However, we have analyzed 48 hours post-MI in this study. Our data demonstrated that after 48 hours postmyocardial infarction there are changes in microglia phenotypes. In particular, we observed the appearance of a dystrophic-like phenotype of microglia mainly in the thalamus and prefrontal cortex. This phenotype of microglia is predictive of a brain malaise that could, in turn, mirror in a malfunction of the neurons. Indeed, dystrophic (senescent) rather than activated microglia seem to be associated with tau pathology and likely precede neurodegeneration in Alzheimer's disease in human [23]. This microglia phenotype is not well characterized yet in terms of the specific marker expression. Recent data in rodents highlighted for the first time dystrophic-like microglia phenotype in transgenic mouse model lacking for the D-Aspartate oxidase, the enzyme responsible of the D-Aspartic acid degradation, that showed high glutamate levels which, in turn, could be in part responsible of those microglia changes [24]. Moreover, a recent report showed a possible microglia change from a reactive to an age-like phenotype with the time in culture [25]. According to the early appearance of dystrophic microglia, we found in the same areas a significant increase of reactive hypertrophic astrocytes. All these changes could be due to a glutamate spillover possibly induced by an overstimulation of afferent fibers after the surgically-induced MI, rather then to the MI per se. Despite this hypothesis, which needs to be verified, it is intriguing that the prolonged exercise prevents these microglia and astrocyte alterations in the prefrontal cortex, thalamus, and prefrontal cortex.

The present study suggests that myocardial infarct may be correlated with a supraspinal synaptic reorganization in which microglia and astrocytes might play a role. Indeed, in our experiments the scale of infarction was strongly correlated with the proportion of dystrophic or activated microglia and hypertrophic astrocytes in the brain. It is well known that, upon activation, morphological cells rearrangements are correlated with the synthesis and secretion of cytokines, by leading critical neuronal activity modifications [14, 26]. Thus, one can speculate that supraspinal nonneuronal cells may significantly contribute to the functional as well as behavioral alterations observed following myocardial infarction. However, further analysis will be necessary to more conclusively evaluate the phenotypical cells profiles induced by MI. In this context, the exercise seems to drive down the possible glial/microglial contribution to the functional alterations associated with myocardium infarct.
